# From ocean to emotion: a pilot study exploring acute mood effects following consumption of a DHA-rich powder compared with placebo in middle-aged Australian men

**DOI:** 10.1017/S0007114524002204

**Published:** 2026-03-28

**Authors:** Jeffery M. Reddan, Sarah Gauci, Lauren M. Young, Greg Kennedy, Renee Rowsell, Anne Marie Minihane, Andrew Scholey, Andrew Pipingas

**Affiliations:** 1 Centre for Mental Health and Brain Sciences, Swinburne University of Technology, Hawthorn, VIC, Australia; 2 Food & Mood Centre, The Institute for Mental and Physical Health and Clinical Translation (IMPACT), School of Medicine, Deakin University, Geelong, VIC, Australia; 3 Norwich Medical School, University of East Anglia, Norwich, UK; 4 Department of Nutrition, Dietetics and Food, Monash University, Notting Hill, VIC, Australia

**Keywords:** *n*-3 PUFA, DHA, Mood, Alertness, Middle-aged, Acute, Nootropic

## Abstract

While there is evidence that long-chain *n*-3 PUFA supplementation benefits mood, the extent to which a single high dose of *n*-3 PUFA can induce acute mood effects has not been examined. The present study investigated whether a single dose of a DHA-rich powder affects self-reported mood in middle-aged males during elevated cognitive demand. In a randomised, double-blind, placebo-controlled trial with a balanced crossover design, twenty-nine healthy males (age M = 52.8 years, sd = 5.3) were administered a powder (in a meal) containing 4·74 g *n*-3 PUFA (DHA 4020 mg; EPA 720 mg) or placebo in random order on two different testing days separated by a washout period of 7 ± 3 d. Participants completed mood assessments before and after completing two cognitive test batteries at baseline and again 3·5–4·0 h following the consumption of the active treatment or placebo. While completion of the cognitive test batteries increased negative mood, differential effects for alertness (*P* = 0·008) and stress (*P* = 0·04) followed consumption of the DHA-rich powder compared with placebo. Although alertness declined when completing the cognitive batteries, it was higher following consumption of the DHA-rich powder compared with placebo (*P* = 0·006). Conversely, stress was lower following consumption of the DHA-rich powder relative to placebo, though this difference only approached significance (*P* = 0·05). Overall, results from this pilot study demonstrate that a single high dose of *n*-3 PUFA may deliver acute mood benefits following elevated cognitive demand in healthy middle-aged males.

Mood is a subjective emotional state that plays an important role in maintaining well-being, which in many people is clinically disordered. Mood disorders such as depression are among the leading causes of disease burden worldwide, accounting for 125·3 million disability-adjusted life years^(1)^ and a point prevalence of 12·9 % within the general population^(2)^. However, this does not account for the burden of ‘subthreshold’ disordered mood within the general population, such as subthreshold depression (approximately 11 % prevalence^(3)^) and generalised anxiety (approximately 4·4 % prevalence^(4)^). Interestingly, one commonly cited reason for the use of complementary medicines, such as dietary supplements, in the general population is to help manage chronic diseases^(5)^, including mental health issues such as disordered mood^(6,7)^. Research from the field of nutritional psychiatry suggests that nutrition plays an important role in maintaining mood and mental well-being^(8)^. There is increasing evidence that adherence to more healthful dietary patterns, such as a Mediterranean-style diet, is associated with a reduced risk of developing poor mental health^(9)^; conversely, evidence indicates that adherence to Western diets high in ultra-processed foods are associated with an increased risk of poor mental health^(10)^. In addition to whole dietary patterns, greater chronic intake of specific nutrients, such as B vitamins, vitamin D and long-chain *n*-3 PUFA (ω-3 PUFA), have been found to be related to a reduced risk of mood disorders^(11–14)^. This suggests a role for dietary interventions, including specific dietary supplements, to benefit mood.

The ω-3 PUFAs – DHA and EPA – are essential for healthy brain function^(15,16)^. Brain tissue is highly enriched in DHA relative to most other organs, with well-defined roles including maintaining cell membrane integrity and function, neuronal plasticity, neurogenesis and the regulation of neuroinflammation and *β*-amyloid clearance^(17)^. EPA has also been shown to be particularly important in glial cell functionality^(18)^. However, because endogenous biosynthesis of these fatty acids is low, it is recommended that they be obtained from external dietary sources. Studies exploring dietary sources of ω-3 PUFA have shown improved mood outcomes and reduced risk of developing mental health disorders with greater consumption^(19)^ but also that supplementation benefits clinically disordered mood^(20–22)^. However, the heterogeneity between clinical trials demonstrates the need for more high-quality randomised controlled trials^(20,21)^.

As indicated above, there is increasing interest regarding the extent to which longer-term dietary behaviours are associated with healthier mood. However, there is also growing interest as to whether dietary interventions are capable of delivering benefits to subjective mood in the hours after ingestion. Indeed, there are data indicating that a single serving/dose of specific foods or dietary bioactives can induce immediate effects on biological processes such as cardiovascular function^(23–25)^, cerebral blood flow^(26,27)^ and inflammation^(28,29)^. It may be that acute effects upon these or other biological processes mediate subsequent acute effects upon subjective mood following a single dose of various dietary extracts or compounds. Examples of dietary extracts shown to benefit mood in the hours following consumption include coffeeberry^(30)^, apple^(26)^, saffron^(31)^ and blackcurrant^(32)^ extracts. Acute mood effects have also been reported following single doses of cocoa flavanols^(33–35)^, flavonoid-rich orange juice^(36)^, wild berry drink^(37)^, decaffeinated coffeeberry^(38)^, green coffee^(39,40)^ and tryptophan-rich hydrolysed protein^(41)^. Various nootropic formulations have also been shown to benefit mood in the hours after a single dose^(42–45)^. Importantly, many of these studies specifically report that these dietary bioactives exerted a mitigating effect upon negative mood change occurring in response to elevated cognitive demand^(34,35,46)^. In a study of 50- to 75-year-old women, a single dose of a multivitamin, mineral and herbal supplement was found to improve overall mood rating as scored by the DASS (depression anxiety stress scale), reduce ratings of stress and increase the rating of calmness measured using a visual analogue scale^(47)^. However, other dietary supplement studies have found no such effects upon acute mood following a single dose^(48–50)^.

To date, there are no published studies examining the acute impact of a single dose of ω-3 PUFA on mood outcomes in the hours following consumption. The paucity of such studies is intriguing, especially given prior evidence that acute mood effects are possible following consumption of a single dose of other nutritional products, but also evidence that ω-3 PUFA supplementation can support healthier mood^(19–22,44,45)^. Therefore, the present study aimed to examine whether a single high dose of ω-3 PUFA, specifically a DHA-rich powder incorporated into a meal, can affect mood in response to elevated cognitive demand in healthy middle-aged males.

## Methods

This pilot trial utilised a double-blind, placebo-controlled balanced crossover design. This was classified as a pilot trial as this is the first to assess the acute impact of a single dose of ω-3 PUFA on mood outcomes; therefore, the sample size and dosage were exploratory in nature. All participants provided written informed consent prior to participation in the study. The study was conducted according to the guidelines laid down in the Declaration of Helsinki, and all procedures involving human subjects/patients were approved by the Swinburne University Human Research Ethics Committee (2018/160). The trial (‘Post-prandial cognitive and vascular effects of a DHA-rich *n*-3 powder in healthy middle-aged males’) was registered with the Australian and New Zealand Clinical Trials Registry (ACTRN12618001160224; www.anzctr.org.au/Trial/Registration/TrialReview.aspx?ACTRN = 12618001160224) and was conducted from July 2018 to April 2021. The primary outcomes from this trial have been previously reported^(24)^. In summary, no significant differential treatment effects were observed for cognitive performance. However, a significant treatment by time interaction was apparent for aortic systolic blood pressure (*F* = 5·95, *P* = 0·022), demonstrating that a reduction in aortic systolic blood pressure (pre-dose to post-dose) was apparent following consumption of the ω-3 PUFA treatment (mean difference = –4·11 mmHg, *P* = 0·004) but not placebo (mean difference = –1·39 mmHg, *P* = 0·12). Furthermore, we observed the expected increase in plasma DHA following the consumption of the DHA-rich powder. Both these findings indicate that acute physiological effects were achievable in the hours after consuming the DHA-rich powder.

### Participants

Male participants aged between 40 and 60 years were recruited from the local community. As this was a pilot study, with cognitive function as the primary outcome^(24)^, recruitment was limited to males, controlling for sex differences to achieve a more homogenous study cohort^(51,52)^. Eligibility criteria included being free from CVD or uncontrolled hypertension (systolic > 160 mmHg and/or diastolic > 90 mmHg), type 1 diabetes or pharmaceutically managed type 2 diabetes, recent head trauma, dementia or cognitive impairment (Mini-Mental State Examination (MMSE) score < 24)^(53)^, a history of neurological conditions (e.g. stroke) and mood or psychiatric disorders, as well as any gastrointestinal, endocrine or bleeding disorders. Participants were also excluded if they were current smokers or taking nicotine in other forms (including the use of nicotine-containing products such as patches, gum or ‘vapes’) or using any dietary supplements or medications expected to influence cognitive function. Consumption of more than one serving of oily fish/seafood per week was also an exclusion criterion (participants were also instructed to avoid consuming fish/seafood the night prior to experimental testing).

**Figure 1.**
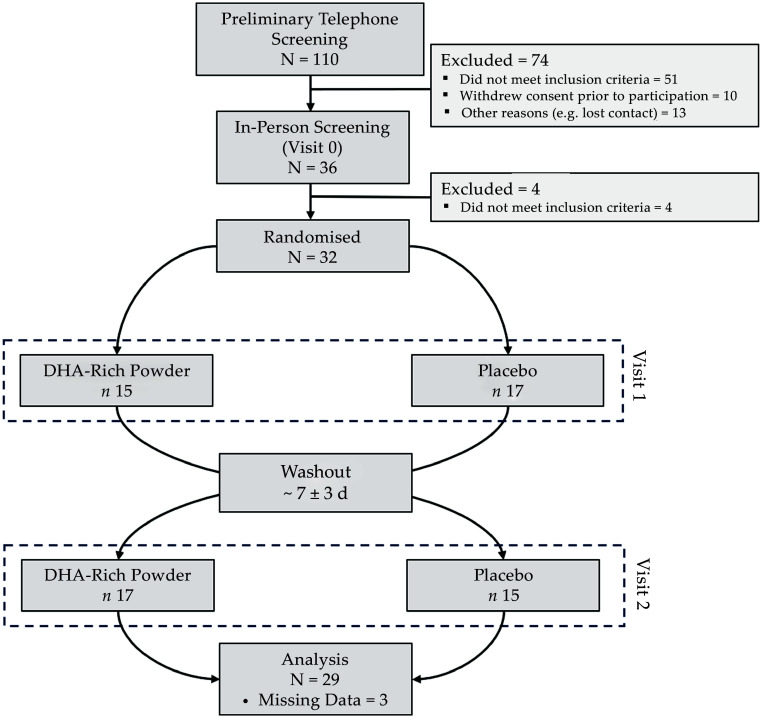
Figure 1 long description. Overview of trial design, participant recruitment and retention.

Overall, 110 possible participants underwent telephone screening, with thirty-six completing subsequent in-person screening at the Centre for Human Psychopharmacology (now Centre for Mental Health and Brain Sciences) lab at Swinburne University of Technology (Melbourne, Australia). Following in-person screening, thirty-two participants were randomised and completed the trial. Randomisation of treatment order was performed by an employee in our research centre not involved with the present study using a computer random number generator. The randomisation key was not accessed by study staff until the formal analysis was complete. Due to missing or incomplete data for three participants, only twenty-nine participants were included in the final analysis. Figure 1 provides an overview of the crossover design of the trial and reports on the flow of participants through the trial.

### Investigational product

The experimental treatment was a microencapsulated tuna oil powder (Driphorm® HiDHA®360), manufactured and supplied by Nu-Mega Ingredients Limited. Each 12 g serving provided 4·74 g ω-3 PUFA (4020 mg DHA and 720 mg EPA)^(24)^. A spray-dried powder containing sunflower oil, matched in appearance to the experimental treatment, was also utilised as a placebo. The powders were added to a single serving (140 g) of vanilla-flavoured Greek yogurt, which was then mixed thoroughly to ensure textural consistency. A single drop of fish oil was added to the placebo to replicate a similar taste and smell as the experimental treatment. Trial staff immediately involved with data collection and analysis did not prepare treatments and were not present during treatment preparation to maintain blinding.

### Inducing cognitive demand

**Figure 2.**
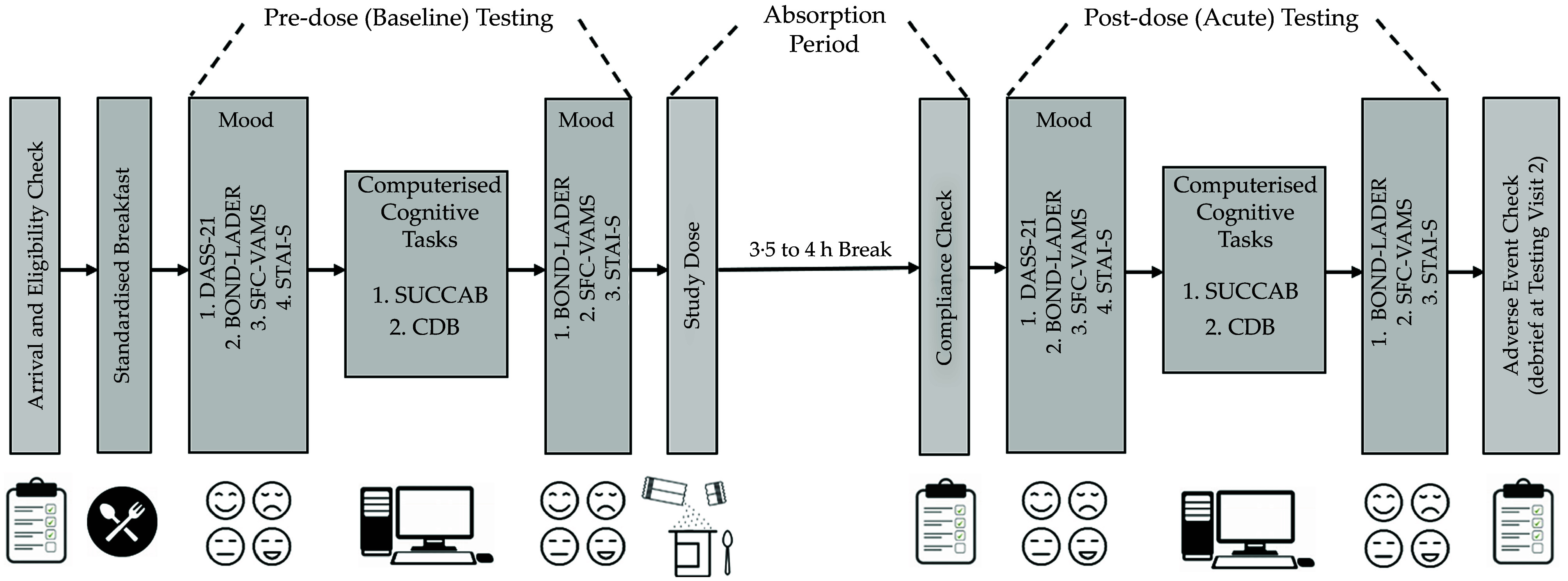
Figure 2 long description. Overview of schedule on experimental testing days. DASS-21, 21-item Depression Anxiety Stress Scale; SFC-VAMS, Stress, Fatigue and Concentration Visual Analogue Mood Scales; STAI-S, State-Trait Anxiety Inventory – State Index; SUCCAB, Swinburne University Computerised Cognitive Assessment Battery; CDB, cognitive demand battery.

Two cognitive batteries were administered consecutively during pre-dose (baseline) and post-dose (acute) testing in order to assess cognitive function. Mood was assessed immediately before and after the completion of these cognitive batteries using visual analogue scales and computerised Likert scales (see Fig. 2).

The first battery, the Swinburne University Computerised Cognitive Assessment Battery (SUCCAB)^(54)^, assessed the primary outcome (i.e. cognitive response time), the results of which have been reported previously^(24)^. The second battery, the cognitive demand battery (CDB)^(35)^, immediately following SUCCAB, is comprised of three tasks (serial 3 subtraction, serial 7 subtraction and rapid visual information processing tasks), which participants completed three times sequentially over a period of approximately 30 min. The CDB was included in the trial to induce workload stress via an increased cognitive load, facilitating a shift towards negative mood (i.e. reduced alertness, elevated stress and increased fatigue)^(34,35,38)^. While the SUCCAB was not specifically designed to negatively affect participant mood (e.g. alertness, stress or fatigue), the battery has been shown in earlier acute mood studies to mentally tax participants^(34)^.

### Subjective state mood assessments

Participants were asked to report their current (i.e. ‘right now’) mood states before and after the two cognitive batteries using visual analogue scales, specifically, the Bond-Lader Visual Analogue Scales and Stress, Fatigue and Concentration Visual Analogue Mood Scales. The Bond-Lader Visual Analogue Scales and Stress, Fatigue and Concentration Visual Analogue Mood Scales, or similar, have been used previously to assess changes in mood following the completion of cognitive tasks and are sensitive to different treatment effects of nutritional interventions^(32,34,39,50)^. The State-Trait Anxiety Inventory – State Index (STAI-S) was also completed.

The Bond-Lader Visual Analogue Scales^(55)^ are comprised of sixteen 100 mm lines anchored at either end by antonyms (e.g. ‘sad’ and ‘happy’). Participants were required to mark the line between these antonyms to indicate their current mood (i.e. are they closer to sad or happy?). The score for each item was determined via the distance in millimetres between the participant’s mark and the negative antonym. Higher scores for each item reflect a greater positive mood. Scores for different items can be combined to describe three factors: alertness, calmness and contentedness. Higher scores for each factor reflect greater alertness, calmness or contentedness.

The Stress, Fatigue and Concentration Visual Analogue Mood Scales consist of four 100 mm lines anchored at either end with the labels ‘not at all’ and ‘very much so’. Participants were instructed to mark each line to reflect the extent to which they felt stressed, mentally or physically fatigued or able to concentrate. Scores for each item were determined by the distance in millimetres between the participant’s mark and the ‘not at all’ label. As such, higher scores reflect greater feelings of stress or fatigue. For the concentration scale, higher scores reflect a greater ability to concentrate.

The STAI^(56)^ was designed to measure general, stable levels of anxiety (trait), as well as fluctuating levels of anxiety (state). The STAI-S consists of twenty items. Participants responded to each item by indicating along a four-point Likert scale the extent to which that item applied to them at that precise moment. Possible responses ranged from ‘not at all’ to ‘very much so’, with the range of possible scores for the STAI-S being 20–80. Higher STAI-S scores reflect greater state anxiety.

### Demographics and participant characterisation

Demographic and anthropomorphic data were collected in person during the initial screening and familiarisation visit (V0, see Fig. 1). Participants were also asked to complete the MMSE^(53)^ to characterise global cognitive function and the 21-item Depression, Anxiety and Stress Scale^(57)^ to characterise mental health at baseline.

### Procedure

Participants were initially screened for eligibility via telephone. Individuals deemed eligible were invited to attend the Centre of Human Psychopharmacology at Swinburne University of Technology to provide written informed consent and finalise eligibility screening. Participant eligibility was confirmed with the completion of additional screening measures (e.g. MMSE) as well as a medical history taken by a trained research nurse. All study procedures were then explained to eligible participants before completing a practice session with the cognitive tasks.

All experimental testing occurred on two separate days. The first was scheduled to occur within 14 d of the in-person screening/familiarisation visit, while the second was scheduled to occur 7 d (± 3 d) after the first testing visit. This was to ensure an appropriate washout for the initial treatment. However, due to changes in participant availability, some participants (a total of seven) had their second testing visit scheduled outside this time (ranging from 11 to 26 d). Importantly, no participants completed the second testing day earlier than specified ensuring adequate washout. All testing was performed in a dedicated human laboratory, which was free from outside distractions. All cognitive and mood assessments were presented to participants using the same size LCD computer monitor (this avoids variance in stimulus size, which for mood assessment would likely impact the magnitude of responses made by participants). All participants arrived at our lab in the morning having been fasting since 22.00 hours the night before (with participants requested to avoid fish/seafood or other foods high in ω-3 fatty acids for dinner the night before, as well as vigorous physical activity and alcohol for at least 12 h prior to the start of testing. Compliance with these criteria was confirmed at the start of each study visit). Following an initial blood sample and cardiovascular assessment^(24)^, participants were provided with a standardised breakfast – a choice of wholemeal toast with jam/vegemite/peanut butter or cereal. Participants were provided the same breakfast the following visit. A standardised meal was also provided 75 min after breakfast (i.e. salad roll with or without ham or chicken) with the study treatment (i.e. DHA-rich powder or placebo mixed in a single 140 g serving of Greek yogurt). The same foods were provided to participants on the second experimental testing day. Participants did not consume any other food or drinks except for plain water on the experimental testing days. Additional details about the testing procedure have been previously reported^(24)^. See Fig. 2 for an overview of experimental testing.

### Analysis

Statistical analyses were conducted using International Business Machines SPSS Statistics for Windows version 26 (IBM Corp.). Baseline data are presented as the mean, standard deviation and range of scores for continuous variables and percentages for categorical variables (see Table 1). Univariate outliers were determined using the lower/upper bound (i.e. 25th and 75th percentile) method^(58)^. The assumption of normality was assessed by examining the skewness/skewness error Z-score for each measure, with a Z-score less than 1·96 indicating a normal distribution^(59)^. If distributional normality was not apparent, commonly employed data transformations (e.g. square root, Log10) were applied, sometimes after the addition of a small constant. Treatment effects upon subjective state mood were examined using a two-way repeated-measures ANOVA. All analyses were performed using data in its original form and repeated following the exclusion of outliers and transformations required for meeting the distributional assumptions of repeated-measures ANOVA. As the current examination is a secondary analysis of measures not designated as the primary outcome of the pilot study from which they were derived^(24)^, no formal power analysis was performed. Statistical significance was set at *P* < 0·05 (two-tailed).

**Table 1.** Participant demographics (Mean values and standard deviations; numbers and percentages) Table 1 long description.

	M	sd	Range
Age (years)	52·8	5·3	42–60
MMSE	29·3	0·9	26–30
Education (years)	16·8	2·2	11–21
	*n*	%	
Highest education achieved, *n* (%)			
Secondary	2	6·9	
Tertiary	19	65·5	
Postgraduate	8	27·6	
Current employment status, *n* (%)			
Student	1	3·4	
Part-time/casual	3	10·3	
Full time	18	62·1	
Retired	4	13·8	
Unemployed	3	10·3	
	M	sd	
BMI (kg/m^2^)	27·4	3·8	21·1–38·6
DASS-21 total score	2·03	2·34	0–8
Depression	0·52	0·99	0–3
Anxiety	0·45	0·69	0–2
Stress	1·07	1·77	0–8
RBC ω-3 PUFA content (as %)			
Total ω-3 PUFA	8·51	1·52	4·33–10·71
DHA	4·54	1·17	2·13–6·68
EPA	0·94	0·24	0·47–1·43
ω-3 Index	5·48	1·29	2·60–7·82

*MMSE*, Mini-Mental State Examination; *DASS-21*, 21-item Depression Anxiety Stress Scale. *n* 29.

## Results

### Demographics

An overview of the demographic information of the participants (*n* 29) is provided in Table 1. In brief, participants were aged between 42 and 60 years (M = 52·8, sd = 5·3). Participant education attainment ranged from 11 to 21 years, with most (*n* 27) having completed tertiary or postgraduate education. Most were working full time (*n* 18) or part-time/casual (*n* 3). The mean participant MMSE score was 29·3 (sd = 0·9). None of the participants had a current history of disordered mood or was taking medications for treating such conditions. Further, the three-factor scores (i.e. depression, anxiety and stress) from the 21-item Depression, Anxiety and Stress Scale (assessed during pre-dose testing at visit 1) were all in the normal range (depression 0–9; anxiety 0–7; stress 0–14). Finally, erythrocyte ω-3 PUFA status of participants in this study is comparable to data from earlier studies with Australian samples (relative % of EPA = 0·43–1·03, DHA = 2·30–5·40, EPA + DHA = 2·91–6·28)^(60)^.

### Change in mood with elevated cognitive demand during pre-dose testing

The purpose of analysing mood data prior to participants having received their scheduled treatment (‘pre-dose’ testing in Fig. 2) was to establish that the completion of the cognitive testing batteries does negatively influence mood. As expected, the completion of the SUCCAB and CDB altered participants’ subjective mood. Specifically, there were significant main effects of time for each mood measure (see Table 2). Alertness, calmness, contentedness and the ability to concentrate were all significantly lower after cognitive testing, while stress and mental and physical fatigue were significantly elevated. Similarly, there was a significant main effect of time for state anxiety (STAI-S), indicating that anxiety increased as a result of completing the cognitive batteries (see Table 2). As expected, prior to participants being administered the DHA-rich powder or placebo, there were no differences in mood changes in response to increased cognitive demand between the two groups (for all interaction effects *P* > 0·05). The results were unchanged following the removal of outliers and/or transforming data to achieve normality. As such, the results presented in Table 2 are those using data in its original form.

**Table 2.** Change in mood with elevated cognitive demand during pre-dose testing (Mean values and standard deviations) Table 2 long description.

Mood	DHA-rich powder						Placebo								
	Pre-cognitive testing		Post-cognitive testing		Mean change	*P*	Pre-cognitive testing		Post-cognitive testing		Mean change	*P*	Main effect of time		
	M	sd	M	sd			M	sd	M	sd			*F*	*P*	Partial η^2^
Alertness	77·7	13·0	57·0	19·7	–20·6	<0·001	77·2	14·6	55·2	21·5	–22·0	<0·001	51·66	<0·001	0·65
Contentedness	82·2	11·3	67·8	17·4	–14·4	<0·001	81·1	12·4	65·5	17·1	–15·6	<0·001	33·45	<0·001	0·54
Calmness	77·6	12·9	62·6	20·0	–15·0	<0·001	76·5	14·1	59·7	15·9	–16·8	<0·001	37·35	<0·001	0·57
Stress	22·7	22·1	36·1	18·1	+13·4	0·004	20·8	20·6	38·4	20·3	+17·6	0·002	16·12	<0·001	0·37
Mental fatigue	17·8	12·3	56·0	21·9	+38·1	<0·001	18·9	15·1	54·3	23·0	+35·4	<0·001	99·71	<0·001	0·78
Physical fatigue	20·1	17·8	39·5	21·1	+19·4	<0·001	19·6	15·2	36·4	20·8	+16·8	<0·001	25·23	<0·001	0·47
Concentration	73·8	19·8	45·6	24·9	–28·2	<0·001	71·1	21·3	43·9	25·4	–27·2	<0·001	52·33	<0·001	0·65
State anxiety	24·2	4·3	32·3	8·1	+8·1	<0·001	24·9	5·8	32·5	10·1	+7·7	<0·001	42·74	<0·001	0·62

All results are from analyses using data in its original form and *n* 29, except *state anxiety*, *n* 27 due to missing data. Analyses repeated following outlier removal and/or data transformation to achieve normality did not alter the direction of effects or significance of findings reported in this table.

### Differential change in mood with cognitive demand during post-dose (4 h) testing

Outliers were identified for alertness, contentedness, stress and physical fatigue. Sample sizes following outlier removal are reported in Table 3 alongside summary statistics for each measure. In addition, transformations were required to normalise all measures except for alertness and contentedness (see Table 4).

**Table 3.** Summary statistics for mood during post-dose (4 h) testing (Mean values and standard deviations) Table 3 long description.

		DHA-rich powder				Mean change	Placebo				Mean change
		Pre-cognitive testing		Post-cognitive testing			Pre-cognitive testing		Post-cognitive testing		
Mood	*n*	M	sd	M	sd		M	sd	M	sd	
Alertness	27	74·4	14·2	63·7	18·8	–10·8	75·0	14·0	55·7	20·9	–19·2
Contentedness	26	82·6	8·6	76·4	10·3	–6·2	83·3	8·7	73·2	13·8	–10·0
Calmness	29	78·2	13·5	67·1	16·8	–11·1	78·3	12·9	63·5	17·5	–14·9
Stress	28	13·0	9·3	30·5	24·0	+17·5	13·9	13·5	37·9	25·7	+24·0
Mental fatigue	29	29·1	21·4	53·1	27·4	+24·0	34·7	26·4	59·9	25·6	+25·2
Physical fatigue	25	21·1	17·2	25·4	14·8	+4·4	22·5	19·3	34·7	20·2	+12·2
Concentration	29	63·7	23·8	45·0	25·3	–18·7	58·6	30·5	44·1	27·8	–14·5
State anxiety	29	24·7	5·5	29·0	8·9	+4·3	25·8	5·9	31·1	8·4	+5·3

All data are in original form. Note that for all measures, the original *n* was 29, except for *physical fatigue* where initially *n* was 28 (one participant had missing data).

**Table 4.** Differential change in mood with cognitive demand during post-dose (4 h) testing (Mean values and standard deviations) Table 4 long description.

Mood	DHA-rich powder						Placebo								
	Pre-cognitive testing		Post-cognitive testing		Mean change	*P*	Pre-cognitive testing		Post-cognitive testing		Mean change	*P*	Time × Treatment Interaction		
	M	sd	M	sd			M	sd	M	sd			*F*	*P*	Partial η^2^
Alertness	74·4	14·2	63·7	18·8	–10·8	0·002	75·0	14·0	55·7	20·9	–19·2	<0·001	8·19	0·008	0·24
Contentedness	82·6	8·6	76·4	10·3	–6·2	<0·001	83·3	8·7	73·2	13·8	–10·0	<0·001	2·54	0·12	0·09
Calmness^a^	6290·4	2001·4	4769·2	2288·6	–1521·2	<0·001	6299·6	1905·0	4321·9	2230·6	–1977·7	<0·001	1·29	0·27	0·04
Stress^a^	1·3	0·2	1·5	0·3	+0·2	<0·001	1·3	0·2	1·6	0·2	+0·3	<0·001	4·55	0·04	0·14
Mental fatigue^a^	6·0	1·7	7·7	1·9	+1·7	<0·001	6·4	1·9	8·2	1·7	+1·8	<0·001	0·06	0·82	0·00
Physical fatigue^a^	5·4	1·4	5·8	1·3	+0·4	0·04	5·5	1·6	6·5	1·5	+1·00	<0·001	3·75	0·07	0·14
Concentration^a,b^	–6·4	1·7	–7·9	1·7	–1·5	<0·001	–6·9	2·2	–7·9	1·8	–1·00	0·002	1·19	0·28	0·04
State anxiety^a,b^	–0·20	0·02	–0·19	0·03	+0·01	0·002	–0·20	0·02	–0·18	0·02	+0·02	<0·001	1·00	0·33	0·03

^a^Transformations required for calmness (X^2^, negative skew), stress (Log10, positive skew), mental fatigue (√, positive skew), physical fatigue (√, positive skew), concentration (−√(max-x), negative skew) and state anxiety (−1/√, positive skew). A constant of 10 was added to stress, mental fatigue, physical fatigue and concentration ability prior to transformation to aid in achieving a normal distribution. ^b^Note that due to the nature of the transformation used, greater values are represented as being closer to 0. Sample size for each analysis indicated in Table 3.

Consistent with changes in mood observed during pre-dose testing, there was a significant main effect of time for each of the mood measures following increased cognitive demand regardless of the treatment participants had consumed on the day: alertness (F(1,26) = 27·08, *P* < 0·001), contentedness (F(1,25) = 29·07, *P* < 0·001), calmness (transformed; F(1,28) = 47·19, *P* < 0·001), stress (transformed; F(1,27) = 53·42, *P* < 0·001), mental fatigue (transformed; F(1,28) = 43·57, *P* < 0·001), physical fatigue (transformed; F(1,24) = 24·55, *P* < 0·001), ability to concentrate (transformed; F(1,28) = 31·54, *P* < 0·001) and state anxiety (transformed; F(1,28) = 21·94, *P* < 0·001). As indicated in Table 4, each of these measures significantly changed as a result of the increased cognitive demand facilitated by the testing batteries regardless of the treatment received (note that due to the nature of the transformations applied to the ability to concentrate and state anxiety data, values that were originally larger are represented as being closer to 0).

**Figure 3.**
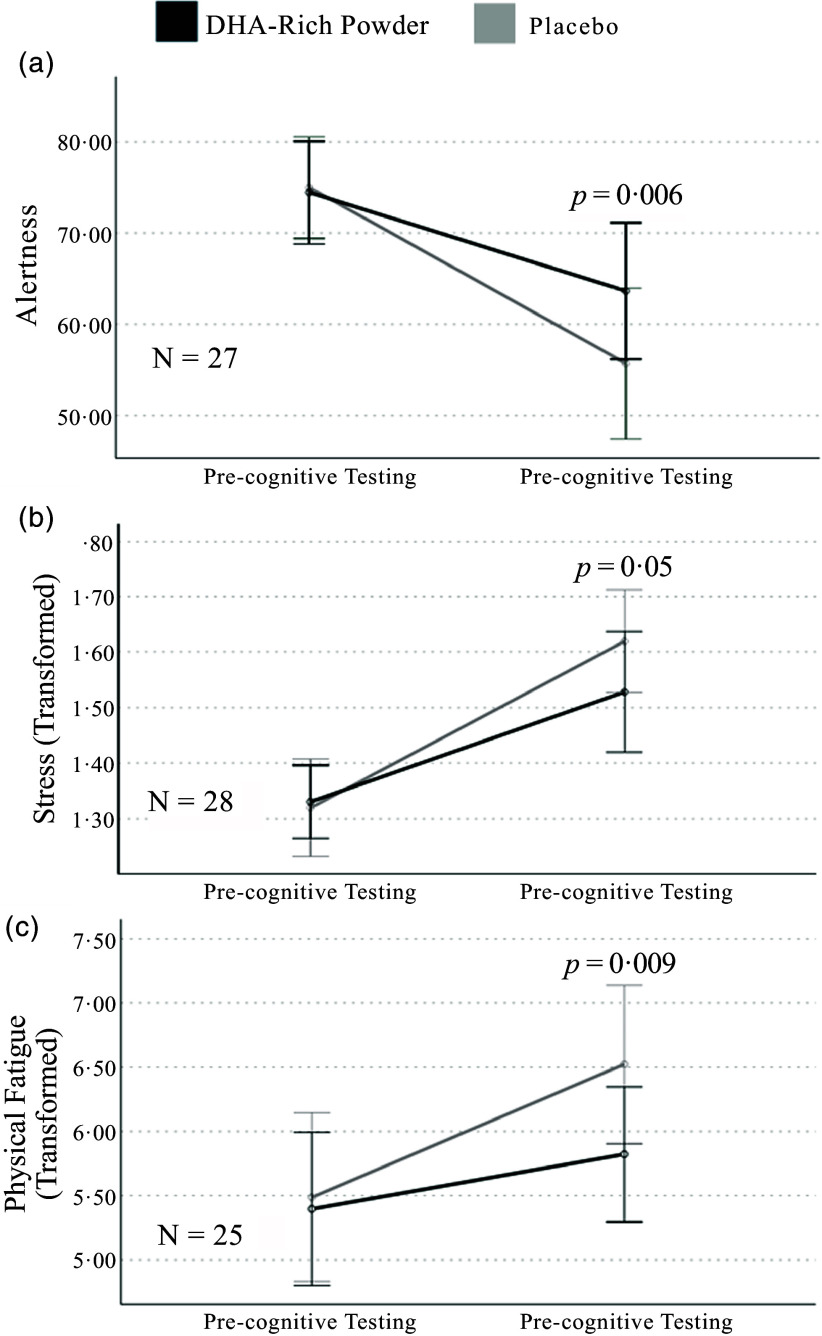
Figure 3 long description. Differential treatment effects on (a) alertness, (b) stress and (c) physical fatigue during post-dose (4 h) testing.

Importantly, a significant differential treatment effect (i.e. interaction) was only apparent for alertness and stress (transformed), while it approached significance for physical fatigue (transformed) (see Table 4). No significant differences in alertness, stress (transformed) or physical fatigue (transformed) were identified across treatment conditions (lowest *P* = 0·71) prior to completing the cognitive batteries and inducing elevated cognitive demand. However, following the completion of the cognitive batteries, alertness was significantly greater for the DHA-rich powder compared with placebo (*P* = 0·006; see Fig. 3(a)). Conversely, while stress (transformed) in response to elevated cognitive demand was lower for the DHA-rich powder, the difference to placebo only approached significance (*P* = 0·05; see Fig. 3(b)). Physical fatigue (transformed) was also significantly lower after completing the cognitive batteries for the DHA-rich powder compared with placebo (*P* = 0·009; see Fig. 3(c)).

## Discussion

The present study examined whether a single dose of a DHA-rich powder, incorporated into a meal, provided acute benefits to mood in middle-aged males following elevated cognitive demand. As expected, the completion of the cognitive batteries significantly altered mood, resulting in reduced self-rated alertness, calmness, contentedness and ability to concentrate, coupled with increased stress and mental and physical fatigue, as well as state anxiety. Significant interaction effects were observed for alertness and stress during post-dose testing, demonstrating that the consumption of the DHA-rich powder altered mood by supporting alertness and buffering the increase in stress in response to elevated cognitive demand. While there was a trend towards a differential treatment effect for physical fatigue, no significant interaction effects were observed for contentedness, calmness, mental fatigue, the ability to concentrate or state anxiety. Thus, data from the present study suggest that the consumption of a high dose of ω-3 PUFA, specifically a high dose of DHA, may represent a viable ‘pre-intervention’ or prophylaxis for mitigating negative mood (i.e. elevated stress, reduced alertness) following enhanced cognitive demand. This may have tangible implications for promoting healthier mental well-being in response to acute periods of elevated cognitive demand such as that experienced in the workplace.

Consistent with results reported here of the acute effects of ω-3 PUFA on alertness, earlier studies have revealed that the consumption of certain food extracts, nutrients and nootropic formulations can benefit subjective alertness following a single dose. For example, in a sample of thirty-two young adults (*n* 6 males), Jackson *et al.* (2020) reported that a single serving of a multi-extract (i.e. beetroot, ginseng and sage) beverage with added apple polyphenols resulted in greater subjective ratings of alertness and reduced fatigue compared with placebo after successive rounds of the CDB (completed 60-, 180- and 360-min post-dose)^(26)^. Other studies investigating acute effects on alertness in the hours following consumption of flavonoids from oranges^(36)^ or blackcurrants^(32)^ have similarly demonstrated a mitigating effect of flavonoids on reduced alertness following elevated cognitive demand. Similar effects have been reported following ingestion of caffeinated coffee in middle-aged and older adults^(40)^, though such effects were also observed following ingestion of decaffeinated coffee delivering a high dose (521 mg) of chlorogenic acid relative to decaffeinated coffee with a lower dose (224 mg). Similar benefits are also reported for subjective stress. One recent crossover study involving healthy young (18–25 years) males demonstrated that a single dose of saffron extract significantly mitigated the rise in subjective stress, as well as anxiety, elicited in response to the Maastricht acute stress test compared with placebo^(31)^. Exploring the acute effects of a single 200 mg dose of l-theanine in healthy young (18–40 years) adults, White *et al.* (2016**)** observed that participants treated with l-theanine exhibited a significantly smaller stress response (i.e. change in mood from start to end of cognitive testing) than placebo, when testing was repeated 1 h post-dose, though no differences were evident 3 h post-dose^(44)^. Acute reductions in stress have also been reported 2 h after a single dose of epigallocatechin gallate (a flavonoid from green tea) in healthy young adults^(45)^. Thus, the present study builds upon an expanding literature demonstrating that a single dose of a nutritional product (i.e. extract or nootropic formulation) is capable of delivering acute mood affects (e.g. alertness, stress) in the hours following consumption, particularly during times of elevated cognitive demand.

While enhanced intake (resulting in a higher status) of ω-3 PUFA over periods of weeks or months has been shown to benefit mood^(21,22,61,62)^, to the best of our knowledge, this is the first study to examine the acute effects of ω-3 PUFA on mood. Due to the limited data on the acute effects of ω-3 PUFA, the results should be interpreted with caution until further evidence is produced. Although this pilot study did not explicitly seek to delineate the mechanisms underlying such effects, it is worth considering possible aetiological mechanisms that could be explored in follow-on randomised clinical trials.

One potential mechanism is an effect on endothelial and overall cardiovascular function. Benefits to central blood pressures following a single dose of the DHA-rich powder in this sample have already been reported^(24)^. There is also evidence from other studies indicating that a meal enriched in ω-3 PUFA (combined DHA + EPA typically 0·90 g to 5·40 g; DHA doses ranged from 0·36 g to 3·24 g; EPA doses ranged from 0·54 g to 2·16 g) may benefit endothelial function in the hours after consumption^(25,63–65)^ and significantly reduced arterial stiffness has been reported 4 h post-dose (significant following 4·16 g DHA but borderline significant following 4·16 g EPA) in a sample of males aged between 35 and 55 years^(25)^. Indeed, in their acute polyphenol study, Jackson *et al.* (2020) reported elevated total Hb using near-infrared spectroscopy, indicative of enhanced cerebral blood flow, following consumption of apple polyphenols, a treatment which also appeared to benefit alertness^(26)^. Jackson *et al.* (2020) suggested that altered cerebral blood flow may have contributed to their findings on subjective mood. Also of relevance is a flavonoid study by Gratton *et al.* (2020), wherein it was reported that a single high dose of cocoa flavanols was capable of benefiting cognitive function, though such effects were only apparent in those who similarly demonstrated enhanced cerebrovascular reactivity and blood flow following the treatment^(27)^. Importantly, an earlier study by Jackson *et al.* indicated that 12 weeks of intake of DHA-rich (1 g DHA) oil, but not EPA-rich (1 g EPA) oil, facilitated enhanced cerebral blood flow (assessed using near-infrared spectroscopy) in young adults while completing cognitive tasks^(66)^. Conversely, another study by Jackson and colleagues failed to identify any significant effect of 6 months of daily intake of 2 g DHA-rich (∼ 0·90 g DHA and 0·13 g EPA) fish oil alone or in combination with a multi-nutrient (containing phosphatidylserine, Ginkgo biloba, folic acid and vitamin B_12_) upon cerebral blood flow in older adults^(67)^. As the above data appear to demonstrate that endothelial and cardiovascular function is modifiable following high dose of ω-3 PUFA intake and that such changes are detectable in the hours following a single dose, it is plausible that a beneficial effect upon vascular function may have contributed to the effect upon subjective mood reported here. This potential mechanism should be explored further in future research.

Another potential mechanism by which ω-3 PUFA consumption could benefit mood, particularly stress, is via the hypothalamic-pituitary-adrenal axis, as indicated via changes in cortisol levels. Noreen *et al.* (2010) reported a ‘tendency for reduced salivary cortisol’ in young and middle-aged (i.e. 18–55 years) adults after 6 weeks of daily supplementation with ω-3 PUFA (1·6 g EPA and 0·8 g DHA) compared with placebo^(68)^. Others have also explored the effects of ω-3 PUFA supplementation on cortisol concentrations in response to enhanced cognitive stress/demand. Delarue *et al.* (2003) demonstrated that an acute mental stressor task enhanced plasma cortisol in healthy young males but that daily ω-3 PUFA supplementation (1·1 g EPA and 0·7 g DHA) mitigated the subsequent rise in plasma cortisol when assessed 3 weeks later^(69)^. Likewise, Barbadoro *et al.* (2013) observed that 3 weeks of daily ω-3 PUFA supplementation (60 mg EPA and 252 mg DHA) in males with alcohol use disorder (participating in a residential rehabilitation programme) significantly reduced daytime salivary cortisol levels (assessed approximately every 4 h) compared with baseline – an effect not observed in those taking the placebo^(70)^. The ω-3 PUFA group also reported a reduction in perceived stress after the 21 d supplementation. Importantly, the authors also assessed changes in salivary cortisol in response to completing a cognitive stressor (i.e. the Trier Social Stress Test) but failed to observe any group differences in the ‘AUC’ or peak cortisol, thereby suggesting no differential treatment effects upon hypothalamic-pituitary-adrenal responsiveness. Giles *et al.* (2015) also reported no significant differences in salivary cortisol change after completing a non-stressful social task and the Trier Social Stress Test in a sample of young adults who either supplemented with ω-3 PUFA or placebo for 3 weeks^(61)^. However, all of the above studies involved longer-term ω-3 PUFA supplementation, not a once-off dose, as in the present work. Therefore, while it has been shown that the cortisol response in response to stressful stimuli is modifiable following a single dose of a nutritional product^(31,44)^, future research is required to determine whether similar changes to the cortisol response are apparent after a single dose of ω-3 PUFA, in particular a high dose of DHA as in the present study. As cortisol was not assessed in the present study, future replication is required to delineate whether a single high dose of ω-3 PUFA is capable of influencing hypothalamic-pituitary-adrenal activity as indicated via changes in cortisol and whether such changes underpin alterations in acute subjective stress.

A particular strength of this study was the use of the SUCCAB and CDB cognitive challenge to induce mood changes, thereby greatly increasing the sensitivity of the assessment. Furthermore, the use of a dedicated human laboratory free from outside distractions provided a controlled environment that may otherwise influence mood (via disengagement with the cognitive tasks). Moreover, mood was assessed using the visual analogue scales immediately before and after the cognitive tasks, which may make them more likely to capture more immediate mood effects than if they were just completed at regular intervals over the testing day.

Nonetheless, the project has several limitations that should be considered when interpreting results. First, the present study sample was relatively small, and it is likely that this reduced the statistical power for detecting significant effects in other measures, such as physical fatigue. A larger sample would no doubt improve statistical power and potentially lead to broader more robust subjective mood effects. Another factor that may have contributed to the limited number of mood measures demonstrating effects in the present study is the restriction of the study sample to males only. Other studies^(34,35,38)^ that have reported differential effects following a single dose of cocoa flavanols upon mental fatigue included male and female participants. It may be that there are sex or gender differences in the extent to which elevated cognitive demand negatively influences mood, and, therefore, the likelihood that acute effects associated with a dietary intervention are detectable. As such, it would be necessary to repeat the present study with a larger representative sample involving males and females to not only assess this possibility but also facilitate greater generalisability of any detected effects.

Future research should investigate whether high dose of ω-3 PUFA can provide acute benefits to mood in clinical populations (e.g. clinical depression or anxiety), who may be more sensitive to the negative influences of cognitive demand and to the benefits of mitigating interventions. While the dosage used in the present study is similar to the ω-3 PUFA profile in a serving of salmon (2·5/100 g)^(71)^, future research should examine whether similar effects are attainable using different dosages as well as fatty acid ratios resembling those attainable from dietary sources. For example, additional research in both clinical and non-clinical samples could assess whether similar mood effects are observed in response to an equivalent high-dose EPA-rich supplement and whether there are differential effects to high-dose DHA. Indeed, a recent meta-analysis^(18)^ reported that treatment with EPA (alone or as the predominant ω-3 PUFA) facilitated greater mood effects than when DHA (alone or as predominant ω-3 PUFA) was administered over longer periods. Considering that the present work is the first to assess the acute effects of any dose of ω-3 PUFA upon subjective mood in response to elevated cognitive demand, it is unknown whether a higher dose of EPA relative to DHA likewise provides benefits to mood. As such, it is possible that the limited effects reported here may become more expansive, or possibly of greater magnitude, if a higher dose of EPA relative to DHA is administered. Therefore, it may be worth replicating the present study with a single high dose of EPA (alone or as the predominant ω-3 PUFA) in order to determine whether similar if not greater effects upon subjective mood are attainable. Future replication directly contrasting higher relative doses of EPA to DHA and vice versa with placebo would also be beneficial for elucidating potentially different mechanisms facilitating acute effects upon subjective mood. Further exploration is needed to determine whether similar effects upon subjective mood are attainable at lower doses, as a lower dose may be more feasible for long-term use.

### Conclusion

The present study revealed that a single high dose of a DHA-rich powder could mitigate the effect of elevated cognitive demand on alertness and stress in healthy middle-aged Australian men. However, further work is required to delineate the potential mechanisms via which a single high dose of ω-3 PUFA may support healthier mood in response to cognitively demanding stimuli and to determine whether acute beneﬁts are similarly achievable in middle-aged females and those with clinical mood disorders.

## Figure 1 Long description

The flowchart begins with preliminary telephone screening of 110 participants, where 74 are excluded for various reasons such as not meeting inclusion criteria or withdrawing consent. The remaining 36 participants undergo in-person screening, with 4 more excluded for not meeting inclusion criteria. This leaves 32 participants who are randomized into two groups: 15 receive DHA-rich powder and 17 receive a placebo. After a washout period of approximately 7 plus or minus 3 days, the groups switch treatments: those who initially received DHA-rich powder now receive the placebo, and vice versa. The final analysis includes 29 participants, with 3 having missing data.

Navigate back to Figure 1.

## Figure 2 Long description

The flowchart outlines the schedule for experimental testing days, divided into three main phases: pre-dose (baseline) testing, absorption period, and post-dose (acute) testing. The pre-dose phase begins with an arrival and eligibility check, followed by a standardized breakfast. Participants then undergo mood assessments using DASS-21, BOND-LADER, SFC-VAMS, and STAI-S scales, followed by computerized cognitive tasks including SUCCAB and CDB. After a 3 to 4-hour break for absorption, participants receive a study dose and have another compliance check. The post-dose phase repeats the mood assessments and computerized cognitive tasks. The final step involves an adverse event check and debriefing at testing visit 2.

Navigate back to Figure 2.

## Figure 3 Long description

The line graph presents data on the effects of DHA-rich powder and placebo on three parameters: alertness, stress, and physical fatigue during post-dose testing. The x-axis represents the time points of pre-cognitive testing and post-cognitive testing, while the y-axis measures the levels of alertness, stress, and physical fatigue. The graph includes three subplots: (a) Alertness, (b) Stress, and (c) Physical Fatigue. In each subplot, two lines represent the DHA-rich powder group and the placebo group. For alertness, the DHA-rich powder group shows a significant decrease from pre-cognitive testing to post-cognitive testing with a p-value of 0.006, while the placebo group also shows a decrease but less pronounced. For stress, the DHA-rich powder group shows a slight increase, while the placebo group shows a more significant increase with a p-value of 0.05. For physical fatigue, the DHA-rich powder group shows a slight increase, while the placebo group shows a more significant increase with a p-value of 0.009. The sample sizes for each subplot are 27, 28, and 25 respectively. All values are approximated.

Navigate back to Figure 3.

## Table 1 Long description

The table presents participant demographics with mean values, standard deviations, and ranges for continuous variables, and percentages for categorical variables. It includes data on age, MMSE scores, education, employment status, BMI, DASS-21 total scores for depression, anxiety, and stress, and RBC omega-3 PUFA content. The table has 15 rows and 4 columns, with column headers labeled M, SD, Range, and n. Notable data points include the mean age of 52.8 years, mean BMI of 27.4 kilograms per square meter, and mean total omega-3 PUFA content of 8.51 percent. The highest education achieved is predominantly tertiary at 65.5 percent, and the majority of participants are employed full-time at 62.1 percent.

Navigate back to Table 1.

## Table 2 Long description

The table presents data on mood changes with elevated cognitive demand during pre-dose testing, comparing DHA-rich powder and placebo groups. It includes pre-cognitive and post-cognitive testing scores for alertness, contentedness, calmness, stress, mental fatigue, physical fatigue, concentration, and state anxiety. The table has 10 rows and 14 columns, with columns for mean and standard deviation values, mean change, P values, and main effect of time. Notable trends include significant decreases in alertness, contentedness, calmness, and concentration, and significant increases in stress, mental fatigue, physical fatigue, and state anxiety after cognitive testing for both groups. The main effect of time is significant for all mood measures.

Navigate back to Table 2.

## Table 3 Long description

The table presents summary statistics for mood measures during post-dose testing, comparing a DHA-rich powder group and a placebo group. It includes data for pre-cognitive and post-cognitive testing phases, with columns for mean values and standard deviations. The mood measures include alertness, contentedness, calmness, stress, mental fatigue, physical fatigue, concentration, and state anxiety. Each row lists the sample size, mean, and standard deviation for both testing phases, along with the mean change. Notable trends include increases in stress, mental fatigue, and physical fatigue for both groups, with the placebo group showing larger changes. Alertness and contentedness decreased in both groups, while calmness showed a reduction. Concentration and state anxiety varied between the groups.

Navigate back to Table 3.

## Table 4 Long description

The table presents data on the differential change in mood with cognitive demand during post-dose testing, comparing DHA-rich powder and placebo groups. It includes pre-cognitive and post-cognitive testing results for various mood metrics such as alertness, contentedness, calmness, stress, mental fatigue, physical fatigue, concentration, and state anxiety. The table has 9 rows and 18 columns, with columns for mean and standard deviation values, mean change, and statistical significance (P values) for both groups. Notable trends include significant changes in alertness, contentedness, calmness, stress, mental fatigue, physical fatigue, concentration, and state anxiety for both groups. The table also includes Time x Treatment Interaction values, highlighting the interaction effects between time and treatment.

Navigate back to Table 4.
